# The Humanistic and Economic Burden Associated with Anxiety and Depression among Adults with Comorbid Diabetes and Hypertension

**DOI:** 10.1155/2018/4842520

**Published:** 2018-10-24

**Authors:** Kimberly Wallace, Xiaohui Zhao, Ranjita Misra, Usha Sambamoorthi

**Affiliations:** ^1^School of Nursing, West Virginia University, Morgantown 26506, USA; ^2^School of Pharmacy, West Virginia University, Morgantown, 26506 WV, USA; ^3^School of Public Health, West Virginia University, Morgantown, 26506 WV, USA

## Abstract

We conducted a retrospective cross-sectional study to estimate the humanistic and economic burden associated with depression and anxiety among adults with comorbid diabetes and hypertension. Pooled data from the 2013 and 2015 Medical Expenditure Panel Survey were used to include adults (≥18 years old) who were alive and diagnosed with both diabetes and hypertension during the observation period. We assessed the humanistic burden with health-related quality of life (HRQoL) and economic burden with the total annual healthcare expenditures. Depending on the presence/absence of depression and anxiety, the study sample was divided into four groups (i.e., no depression/anxiety, depression only, anxiety only, and depression and anxiety). Multivariable regression analyses were used to evaluate the associations between the depression/anxiety categories and disease burden measures. The incremental burden associated with depression and/or anxiety was estimated with the counterfactual recycled prediction. Of the 4560 adults with comorbid diabetes and hypertension, 13.2% reported depression only, 8.7% reported anxiety only, and 7.7% reported both. Results from adjusted analyses indicated that the presence/absence of depression and anxiety was associated with significantly poorer HRQoL, especially on the mental component. Having either depression or anxiety corresponded to reduced mental component summary scores by more than four points. The reduction was as high as 10.35 points when both conditions occurred. Comparing to adults without depression or anxiety, the per-capital incremental annual healthcare expenditures were $4607 for the depression group, $2481 for the anxiety group, and $8709 for adults with both conditions. Furthermore, adults with depression and anxiety were 58% more likely to spend at least 10% of annual household income on healthcare as compared to those with neither the conditions. Our results highlight the needs for integrating cost-effective mental health services into diabetes management to improve the HRQoL and reduce healthcare costs for adults with comorbid diabetes and hypertension.

## 1. Introduction

Comorbid chronic conditions can post an enormous challenge in diabetes care. Hypertension is one of the most common comorbid conditions due to a considerable overlap of risk factors [1]. Approximately 75% of individuals diagnosed with diabetes have concomitant hypertension, representing nearly 23 million adults in the United States (US) [1, 2]. Individuals with multiple chronic conditions usually have impaired mental health [3]. Like many other chronic conditions, diabetes and hypertension are independently associated with a higher prevalence of mental conditions, particularly depression and anxiety [4]. The prevalence of depression and anxiety ranges from 15% to 35% among individuals with diabetes [5, 6] and 22% to 56% among those with hypertension [7, 8].

The presence of depression and/or anxiety can impose substantial disease burdens on adults with comorbid diabetes and hypertension as they ranked amongst the top ten causes of disease burden in the 2013 Global Burden of Disease Study [9]. The presence of either the condition can complicate the course of diabetes and hypertension by causing increased inflammation, unhealthy lifestyles, and poor adherence to treatments [10]. Furthermore, depression and anxiety can lead to significant functional impairments, resulting in poor quality of life [11, 12]. Results from several observational studies have indicated that depression and anxiety manifest themselves in multiple facets of the quality of life for individuals with diabetes, including functional, cognitive, and emotional domains [13–15].

The impairment in quality of life also represents a considerable economic burden on patients, their family members, and the whole healthcare system due to high use of healthcare services. Diabetes is one of the most expensive chronic conditions in the US, with an estimated medical expenditure of $237 billion in 2017 [16]. Based on the finding of a recent study, the national burden of comorbid diabetes and hypertension could exceed $350 billion [17]. Depression and anxiety have been indicated with higher risks of diabetes complications and heart diseases, leading to excess costs from more physician office visits, emergency room or inpatient admissions, and prescription consumption [18, 19].

Depression, anxiety, diabetes, and hypertension are closely linked and affect a substantial proportion of the US population. Individuals that carry more than one, or even all four conditions, face unique challenges regarding access to the healthcare resources and the emotional state to achieve optimal treatment goals. Evaluating the burden of mental comorbidities has indispensable roles in achieving these goals because it helps direct resource allocation for policymakers. Like diabetes and hypertension, depression and anxiety also have substantial overlap in occurrence [20]. Although previous studies have evaluated the impact of depression and anxiety on individuals with diabetes or hypertension separately, no study has comprehensively quantified the effect of either of the condition alone. Furthermore, researchers have reported that treating comorbid depression and anxiety could be more challenging than managing each condition alone [21]. Research attention on comorbid depression and anxiety is therefore warranted to help understand the unmet needs in providing care to patients with such complex conditions. Therefore, the objective of this study is to estimate the humanistic and economic burden of depression and/or anxiety among adults with comorbid diabetes and hypertension.

## 2. Materials and Methods

### 2.1. Study Design

This study utilized a retrospective cross-sectional design.

### 2.2. Data Source

We pooled data from the 2013 and 2015 Medical Expenditure Panel Survey (MEPS). The MEPS is a nationally representative survey of noninstitutionalized adults. It collects information on demographics and various facets of healthcare, including medical conditions, access to care, health-related quality of life (HRQoL), and medical expenditures. The MEPS employs a panel design, in which participants were followed for two years. As recommended by MEPS, we utilized alternate years to avoid duplicate observations of the same participant [22].

### 2.3. Study Sample

Our descriptive sample consisted of 4560 adults (aged 18 or older) who were alive and diagnosed with both diabetes and hypertension during the study period (2013 and 2015). Diabetes and hypertension were identified from the full-year consolidated file based on queries regarding the diagnosis of specific conditions or medical condition files using the clinical classification codes (49–50 for diabetes, 98–99 for hypertension). These conditions were reported by households, recorded by professional coders, and then converted into clinical classification codes by MEPS researchers. Because the MEPS only collects data on HRQoL through self-administered questionnaires (SAQ), we further restricted our sample to adults who were eligible for SAQ to analyze humanistic outcomes. For economic outcomes, the analytic cohort only included adults with positive expenditures.

### 2.4. Dependent Variables

#### 2.4.1. Humanistic Outcome: Health-Related Quality of Life (HRQoL)

MEPS measures HRQoL with the 12-item short-form health survey version 2 (SF-12v2). The SF-12v2 is a widely used generic measure for HRQoL in health services research. MEPS researchers combined, scored, and weighted the responses to create physical component summary (PCS) and mental component summary (MCS) scores. The scores range from 0 to 100, with higher scores indicating better HRQoL related to physical and/or mental health [23].

#### 2.4.2. Economic Outcomes: Total Annual Healthcare Expenditures

The MEPS is considered as the most complete source of data on healthcare expenditures. Healthcare expenditures are measured as any payment to healthcare providers (i.e., hospitals, outpatient facilities, private practices, and long-term care facilities) for healthcare services (i.e., inpatient, outpatient, prescription, dental, vision, and home health services). Payers include patients or their families paying for a service out-of-pocket as well as third-party payers (Medicare, Medicaid, private insurance, Veterans Administration, Tricare, HMOs, etc.). Total annual per-person healthcare expenditures were calculated as the sum of all medical payments from all payers. We conducted separate analyses on total expenditures, those paid by third parties and out-of-pocket (OOP) spending from patients. Third-party expenditures were calculated as the difference between total expenditures and OOP spending. All expenditures were adjusted to 2015 US dollars using the consumer price index for medical services from the Bureau of Labor Statistics.

#### 2.4.3. Economic Outcomes: OOP Spending Burden

The economic burden from OOP spending was measured as the percentage of annual household income spent on healthcare as OOP payments [24]. We used a conventional cut-off of 10% to define high burden [25].

### 2.5. Key Independent Variable

#### 2.5.1. Depression/Anxiety Categories

We identified depression and anxiety from the medical condition files using the clinical classification code of “657” and “651.” A variable with four categories (depression and anxiety, depression only, anxiety only, and no depression/anxiety) was created based on the presence/absence of depression and anxiety.

### 2.6. Other Independent Variables

We selected other independent variables under the guidance of the Andersen's behavioral model [26], in which an individual's use of healthcare services and associated outcomes is considered as a function of predisposing factors, enabling factors, need factors, personal health practices, and external environment. We included sex (male/female), race/ethnicity (Whites/African-American/Latino/other racial minorities), and age (18–39/40–54/55–64/65–74/75+) as predisposing factors. Enabling factors were comprised of marital status (married/not married), education (less than high school/high school/more than high school), family poverty status (poor/near poor/middle income/high income), health insurance (private/public/uninsured), and prescription insurance status (yes/no). Need factors included having a chronic condition (yes/no) other than diabetes and hypertension from a list of eight conditions (asthma, arthritis, cancer, chronic obstructive pulmonary disease, heart disease, and stroke), perceived physical and mental health status (excellent/very good, good, and fair/poor), and pain interference (mild/no, moderate, and a lot/extreme). Personal health practice factors included obesity (yes/no), smoking status (current smoker/others), and physical activities (five times or more/week, less than five times/week). We also used geographic region (northeast/midwest/south/west) of residency to account for variations due to the external environment.

### 2.7. Statistical Analyses

We used chi-square tests to determine statistically significant differences across study groups for categorical variables and Student's independent *t*-tests for continuous variables. Multivariable regression analyses were used to examine the association between the depression/anxiety categories and disease burden measures. Ordinary least square regressions were employed to analyze MCS and PCS scores. Logistic regressions were used for binary outcomes such as OOP spending burden. To overcome common challenges associated with modeling expenditures (e.g., high positive skewness and heteroscedasticity), we used generalized linear models with a log link function and gamma family distribution for expenditure outcomes [27].

To estimate excess healthcare expenditures contributed by depression and/or anxiety, we utilized counterfactual recycled prediction. This technique is preferable because, by creating counterfactual scenarios, it allows for adjustment for differences in characteristics across all groups (i.e., depression and anxiety, depression only, anxiety only, and neither depression nor anxiety) [28]. To account for the complex survey design of the MEPS, we utilized the survey procedures in the statistical analysis software (SAS) version 9.4 (Cary, NC, USA) and STATA 14. As recommended by MEPS researchers, we computed annualized weights by dividing personal weights by the number of years pooled (two, in our study) [29]. Annualized SAQ weights were used for analyzing MCS and PCS.

## 3. Results

### 3.1. Description of Study Sample

The study sample was almost evenly female (50.1%) and male (49.9%). The majority were white (62%), aged greater than 55 (76%), with multimorbidity (73%), and adults who had at least a high school education (80%). Twenty-seven percent of individuals considered themselves as having excellent or very good physical health while 48% reported having excellent or very good mental health (Table 1).

Within our sample, approximately one-fifth had either depression (13.2%) or anxiety (8.7%), and 7.7% had both conditions. We observed significant differences in the prevalence of depression and/or anxiety across all predisposing factors, enabling factors, need factors, personal health practices, and external environment with the exception for education. For example, females with comorbid diabetes and hypertension reported a significantly higher rate of depression and anxiety, either alone or together, as compared to their male counterparts. Similar patterns were observed for adults with public insurances relative to private insurances or no insurance, adults reporting multimorbidity in addition to diabetes and hypertension versus those without additional chronic conditions, and adults with severe pain interference as compared to those with mild or no pain interference (Table 1).

### 3.2. Humanistic Outcomes: Physical and Mental Component Summary Scores (PCS and MCS)

Adults with depression and/or anxiety reported significant lower health scores, both physically and mentally, as compared to those with neither the condition. Across the four groups, adults with both depression and anxiety reported the lowest MCS scores while those with depression had the lowest PCS scores (Table 1). However, after adjusting for all other covariates, we only observed significant differences in MCS scores. Results from adjusted analyses indicated that, compared to adults with neither depression nor anxiety, the MCS scores were 10.35 points lower for those with depression and anxiety and 7.67 and 4.88 points lower for those with either depression or anxiety, respectively (Table 2).

### 3.3. Economic Outcomes: Total Annual Healthcare Expenditures

Adults with depression and/or anxiety also had significantly higher annual healthcare expenditures than those with neither the condition (Table 3). Adults with depression and anxiety had the highest expenditures at $28,832 per person per year, followed by those with either depression ($19,648) or anxiety ($16,990). After adjusting for all other factors that may influence healthcare expenditures, results from recycled predictions showed that the annual per-person mean healthcare expenditures were $20,963 (95% confidence interval (CI): $20,581–$21,329) for those with both depression and anxiety and $16,861 (95% CI: $16,533–$17,155) and $14,735 (95% CI: $14,466–$14,992) for those with either depression or anxiety. The expenditures in these three groups were all significantly higher than the group with neither condition, with an incremental expenditure of $8709 (95% CI: $8550–$8861), $4607 (95% CI: $4523–$4687), and $2481 (95% CI: $2436–$2525), respectively (Table 3).

Similar results were found for analyses on third-party expenditures and out-of-pocket (OOP) spending (Table 3). For instance, third-party payers spent an excess of $9132 (95% CI: $8956–$9304) annually among adults with both depression and anxiety than those with neither condition. Furthermore, the former group also had $399 (95% CI: $393–$406) more OOP spending than the latter group.

### 3.4. Economic Outcomes: OOP Spending Burden

We found significantly higher percentages of adults with depression and/or anxiety bearing a high OOP spending burden (i.e., spending 10% or more of income on healthcare) than those without these two conditions (Table 4). Specifically, approximately one-third (34.0%) of those with both depression and anxiety had high OOP burden versus 18.3% of those with neither the conditions. Results from unadjusted and adjusted logistic regressions consistently indicated that adults having depression and anxiety were more likely to suffer from high OOP burden as compared to those with neither the conditions. However, we did not observe any significant association between either condition alone (depression or anxiety) and high OOP burden after adjusting for all the predisposing factors, enabling factors, need factors, personal health practices, and external environment (Table 4).

## 4. Discussion

This study comprehensively evaluated the humanistic and economic burden associated with depression and anxiety, alone and together, among a nationally representative sample of adults with comorbid diabetes and hypertension. Our results revealed that a substantial proportion of adults with comorbid diabetes and hypertension suffered from depression and/or anxiety. Some subgroups such as females, individuals with low socioeconomic status, current smokers, and those with severe pain were more vulnerable than others to be affected. Adding regular check-ups for mental health to diabetes management for these subgroups may help prevent the development of depression or anxiety.

Our study findings showed that depression and anxiety, either alone or together, were associated with poorer quality of life relative to mental health, but not physical health, among adults with comorbid diabetes and hypertension. It is expected that individuals with mental health diagnoses perceived poorer mental health. Although previous literature suggested that depression could impact physical health [30], we did not observe such association after adjusting for all the predisposing factors, enabling factors, need factors, personal health practice, and the external environment. We speculated that adults with diabetes and hypertension perceived the role limitations and impaired functional status affected by emotional problems rather than physical sufferings. It is also possible that they received more medical attention to physical symptoms than mental problems from healthcare providers. Results from the National Comorbidity Survey Replication indicated that 60% of adults with a recent mental health condition did not receive care from healthcare professional [31]. Removing barriers to seeking mental healthcare (i.e., mental health stigma) and increasing access to mental health services are pivotal to improve the quality of life for patients with comorbid diabetes and hypertension.

In addition to impairments in quality of life, adults with comorbid diabetes and hypertension also born excess economic burden from depression and anxiety, especially when both conditions occurred. Li and colleagues reported that almost one-fourth of adults with diabetes spent considerable proportion (>10%) of family income for healthcare [32]. The presence of comorbid depression and anxiety may expose more diabetes patients and their families to financial difficulties. Such excess spending burden may come from paying for behavioral health interventions, which have relatively low reimbursement rates. Lifting restrictions and increasing reimbursement rates for behavioral health services can enable more diabetes patients to afford the care needed for their overall well-being. Not only patients but also healthcare payers such as Medicare, Medicaid, and private insurance companies have significantly higher costs due to depression, anxiety, and the combination of these two. We estimated that depression and anxiety together accounted for an excess of $14.3 billion medical expenditures (2015 USD) for third-party payers annually. The new payment model for behavioral health services implemented by the Centers for Medicare and Medicaid Services may be promising in reducing costs for both patients and payers [33].

Like any other research study, our findings should be interpreted along with the study strengths and limitations. We believe this to be the first study that comprehensively examined the humanistic and economic burden of depression and anxiety among adults with comorbid diabetes and hypertension in a nationally representative sample. Also, our study utilized data that allows for adjustment of a comprehensive list of confounders. The recycled predictions provide realistic estimates of healthcare expenditures for a typical diabetes patient. Such estimates can be better comprehended by policymakers than parameter estimates from conventional regression analyses. However, there are limitations as well. First, the study results may subject to recall and social desirability bias due to the self-reported nature of the MEPS data. Second, there might be misclassification bias from undiagnosed depression/anxiety, leading to underestimation of the disease burdens. Third, the diagnostic codes and clinical classification codes provided by the MPES do not allow us to further examine the burden of anxiety and depression on different types of diabetes and/or hypertension. Also, we could not evaluate the duration and severity of diabetes and hypertension from MEPS data. Future studies considering these factors are warranted to confirm our findings. Finally, the cross-sectional design of our study may limit the ability to demonstrate causality.

## 5. Conclusions

In summary, the present study indicated that depression and anxiety, especially the combination of these two conditions, are associated with significant impairments in quality of life and excess economic burden for patients with comorbid diabetes and hypertension. The burden estimates can be used as benchmarks to evaluate future diabetes management programs. Our findings highlight the needs for timely screening and early interventions that could help prevent anxiety and/or depression among adults with comorbid diabetes and hypertension to reduce their clinical, humanistic, and economic burden. Furthermore, it is necessary to integrate mental healthcare into diabetes management to improve the quality of life for adults with comorbid diabetes and hypertension. Innovative payment models that encourage collaboration among primary care providers, diabetes specialists, and mental health professionals are needed to reduce healthcare costs for both patients and healthcare payers.

## Figures and Tables

**Table 1:** Description of study sample by depression and anxiety categories among adults with comorbid diabetes and hypertension, using pooled data from the 2013 and 2015 Medical Expenditure Panel Survey.

	All		Depression and anxiety		Depression only		Anxiety only		No depression/anxiety	
	*N*	Wt%	*N*	Wt row%	*N*	Wt row%	*N*	Wt row%	*N*	Wt row%	*p* value	sig
All	4560	100.0	309	7.7	561	13.2	366	8.7	3324	70.4		

Predisposing factors												
Sex											<0.001	^∗∗∗^
Female	2477	50.1	227	10.9	368	16.5	227	10.3	1655	62.3		
Male	2083	49.9	82	4.4	193	10.0	139	7.0	1669	78.6		
Race/ethnicity											<0.001	^∗∗∗^
White	1658	61.6	163	9.2	237	14.6	173	10.4	1085	65.8		
African-American	1267	16.1	51	4.0	140	11.1	90	6.3	986	78.6		
Latino	1210	14.4	80	6.8	156	13.2	87	6.4	887	73.6		
Others	425	7.9	15	4.5	28	6.9	16	4.2	366	84.4		
Age groups											<0.001	^∗∗∗^
18–39 years	224	4.4	21	10.5	30	15.5	23	8.7	150	65.3		
40–54 years	1016	19.9	96	10.3	121	13.2	77	8.3	722	68.1		
55–64 years	1304	26.8	98	8.6	191	16.0	98	8.8	917	66.6		
65–74 years	1165	28.6	65	7.3	152	13.8	92	8.2	856	70.8		
75 years or older	851	20.3	29	3.7	67	8.4	76	9.4	679	78.5		

Enabling factors												
Marital status											<0.001	^∗∗∗^
Married	2352	57.5	110	5.7	265	12.9	170	8.0	1807	73.3		
Not married	2208	42.5	199	10.3	296	13.6	196	9.5	1517	66.6		
Education level^†^											0.186	
<High school	1301	19.1	74	6.0	158	11.4	106	9.3	963	73.3		
High school	1447	34.2	110	8.9	174	12.9	126	9.2	1037	69.0		
>HS	1751	46.7	121	7.5	223	14.2	131	8.1	1276	70.2		
Poverty status^‡^											<0.001	^∗∗∗^
Poor	1014	13.5	100	11.5	165	15.6	95	9.6	654	63.2		
Near poor	1174	22.7	88	9.4	138	13.2	105	10.3	843	67.1		
Middle income	1321	30.5	77	7.6	152	13.7	92	7.0	1000	71.6		
High income	1051	33.3	44	5.0	106	11.8	74	8.6	827	74.6		
Insurance coverage											<0.001	^∗∗∗^
Private	2119	57.0	100	5.8	230	13.2	161	8.3	1628	72.7		
Public	2067	37.3	193	10.8	294	14.0	190	9.8	1390	65.4		
Uninsured	374	5.7	16	6.1	37	9.1	15	4.6	306	80.1		
Prescription insurance											0.020	^∗^
Yes	1670	44.7	79	5.9	182	13.8	128	8.6	1281	71.7		
No	2890	55.3	230	9.1	379	12.8	238	8.7	2043	69.4		

Need factors												
Perceived physical health											<0.001	^∗∗∗^
Excellent/very good	1037	26.7	36	4.3	85	9.2	69	8.0	847	78.5		
Good	1642	38.2	81	6.1	160	11.3	117	8.1	1284	74.6		
Fair/poor	1881	35.1	192	11.9	316	18.4	180	9.8	1193	59.8		
Perceived mental health											<0.001	^∗∗∗^
Excellent/very good	1979	48.2	56	3.8	135	8.0	124	7.6	1664	80.6		
Good	1702	35.8	103	7.3	228	15.3	134	8.7	1237	68.6		
Fair/poor	879	16.0	150	20.1	198	24.5	108	11.6	423	43.8		
Multimorbidity											<0.001	^∗∗∗^
Yes	3179	72.6	198	8.6	369	15.3	212	8.8	1729	67.3		
No	1381	27.4	104	6.3	180	10.4	146	8.3	1525	75.0		
Pain interference											<0.001	^∗∗∗^
None/little	2240	50.5	95	5.5	191	9.7	143	7.6	1811	77.3		
Moderate	757	17.5	58	8.4	103	14.4	64	8.6	532	68.6		
A lot/extreme	1204	25.0	136	12.1	228	20.2	132	11.2	708	56.6		
Not reported	359	7.1	20	5.9	39	11.3	27	7.5	273	75.3		

Personal health practice												
Obesity^†^, ^⁋^											<0.001	^∗∗∗^
Yes	2508	57.3	198	8.6	369	15.3	212	8.8	1729	67.3		
No	1955	42.7	104	6.3	180	10.4	146	8.3	1525	75.0		
Current smoker											<0.001	^∗∗∗^
Yes	578	12.2	77	12.8	103	21.0	49	7.7	349	58.5		
No	3553	79.2	214	7.1	415	12.4	281	8.7	2643	71.7		
Not reported	429	8.6	18	5.5	43	9.7	36	9.1	332	75.7		
Exercise ≥ 5 times/week^†^											<0.001	^∗∗∗^
Yes	1584	34.7	77	6.1	154	10.1	99	6.6	1254	77.2		
No	2938	65.3	229	8.5	402	14.9	266	9.8	2041	66.8		

External environment												
Region											0.027	^∗^
Northeast	740	17.2	76	11.1	99	12.7	56	8.1	509	68.0		
Midwest	800	22.0	69	8.9	106	13.0	80	10.2	545	67.9		
South	2011	42.6	109	5.7	250	13.9	166	8.7	1486	71.7		
West	1009	18.3	55	7.6	106	12.4	64	7.2	784	72.8		

Note: study sample included adults (≥18 years old) with comorbid diabetes and hypertension who were alive in the observation year (2013/2015). Wt%: weighted percentages; Wt row%: weighted row percentages; Sig.: statistical significance level. ^†^Some groups with missing data were not reported here due to small cell size (<10). ^‡^Poverty status was calculated from the annual family income and family composition using the federal poverty line (FPL). Poor was defined as <100% FPL; near poor was defined as >100% FPL and <199% FPL; middle income was defined as >200% FPL and <399% FPL; high income was defined as >400% FPL. ^⁋^Obesity was defined as body mass index ≥ 30.0. ^∗^*p* < 0.05; ^∗∗^0.001 < *p* < 0.01; ^∗∗∗^*p* < 0.001.

**Table 2:** Unadjusted and adjusted coefficients for depression and anxiety categories from ordinary least square (OLS) regressions on physical component summary (PCS) and mental component summary (MCS) scores among adults with comorbid diabetes and hypertension, using pooled data from the 2013 and 2015 Medical Expenditure Panel Survey (MEPS).

Unadjusted means and SE				
	PCS		MCS	
	Mean (SE)	*p* value	Mean (SE)	*p* value
Depression and anxiety	37.27 (0.96) ^∗∗∗^	<0.001	40.45 (0.94) ^∗∗∗^	<0.001
Depression only	37.02 (0.97) ^∗∗∗^	<0.001	43.76 (0.71) ^∗∗∗^	<0.001
Anxiety only	38.38 (1.04) ^∗^	0.016	46.96 (0.78) ^∗∗∗^	<0.001
No depression/anxiety (reference group)	40.92 (0.34)		52.8 (0.23)	

Parameter estimates and SE from multivariable OLS regressions^†^				
	PCS		MCS	
	Beta (SE)	*p* value	Beta (SE)	*p* value
Depression and anxiety	−0.68 (0.87)	0.432	−10.35 (0.91) ^∗∗∗^	<0.001
Depression only	−1.37 (0.83)	0.098	−7.67 (0.66) ^∗∗∗^	<0.001
Anxiety only	0.01 (0.80)	0.987	−4.88 (0.74) ^∗∗∗^	<0.001
No depression/anxiety (reference group)				

Note: the analytic sample consisted of adults (>18 years) with comorbid diabetes and hypertension who were alive and eligible for the self-administered questionnaires of MEPS in the observation year (2013/2015). SE: standard error of the mean; Beta: parameter estimates from multivariable OLS regressions. ^†^Covariates included in the multivariable OLS regressions included predisposing factors (sex, age groups, and race/ethnicity), enabling factors (marital status, education level, poverty status, health insurance coverage, and prescription drug insurance coverage), enabling factor (presence/absence of other chronic conditions, pain interference), personal health practice (obesity, smoking status, and exercise level), and external environment (region). Pain interference was not included in the regression on PCS scores because it was used in the computation of PCS scores with heavy weights. ^∗^*p* < 0.05; ^∗∗^*p* < 0.01; ^∗∗∗^*p* < 0.001.

**Table 3:** Unadjusted and adjusted annual per-person mean healthcare expenditures (2015 US$) by depression and anxiety categories among adults with comorbid diabetes and hypertension, using pooled data from the 2013 and 2015 Medical Expenditure Panel Survey.

	*N*	Unadjusted mean	Adjusted mean^†^	Adjusted incremental^†^
		Mean (SE), $	Mean (95% CI)^‡^, $	Mean (95% CI)^‡^, $
All payers				
Depression and anxiety	309	28,832.15 (5963.66) ^∗∗^	20,962.68 (20,580.69–21,328.80)	8708.81 (8550.11–8860.90)
Depression only	559	19,648.34 (1,542.8) ^∗∗∗^	16,860.66 (16,553.42–17,155.13)	4606.78 (4522.84–4687.24)
Anxiety only	366	16,990.31 (2008.43) ^∗∗^	14,735.14 (14,466.63–14,992.49)	2481.26 (2436.05–2524.60)
No depression/anxiety	3256	11,543.4 (448.52)	12,253.88 (12,030.58–12,467.89)	(reference group)

Third-party payers				
Depression and anxiety	309	27,009.6 (5928.43) ^∗∗^	20,396.77 (20,003.49–20,782.35)	9132.19 (8956.11–9304.83)
Depression only	559	17,844.71 (1436.02) ^∗∗∗^	15,659.59 (15,357.65–15,955.62)	4395.01 (4310.27–4478.09)
Anxiety only	366	15,236.58 (1996.7) ^∗^	13,273.26 (13,017.34–13,524.18)	2008.69 (1969.96–2046.66)
No depression/anxiety	3256	10,420.07 (445.14)	11,264.58 (11,047.38–11,477.53)	(reference group)

Out-of-pocket				
Depression and anxiety	309	1822.54 (207.53) ^∗∗∗^	1382.75 (1361.19–1405.12)	399.31 (393.08–405.77)
Depression only	559	1803.64 (236.02) ^∗∗^	1364.25 (1342.98–1386.32)	380.81 (374.88–386.98)
Anxiety only	366	1753.73 (231.31) ^∗∗^	1312.70 (1292.23–1333.94)	329.26 (324.12–334.59)
No depression/anxiety	3256	1123.33 (41.17)	983.44 (968.10–999.35)	(reference group)

Note: the analytic sample consisted of adults (>18 years) with comorbid diabetes and hypertension who were alive and had positive expenditures in the observation year (2013/2015). All the expenditures were converted to 2015 US dollars using the consumer product index from the US Bureau of Labor Statistics. SE: standard error of the mean. ^†^The adjusted annual per-person mean/incremental healthcare expenditures were obtained from recycled predictions based on the estimates of generalized linear model (GLM) with log link function and gamma distribution. Covariates adjusted in the GLM included predisposing factors (sex, age groups, and race/ethnicity), enabling factors (marital status, education level, poverty status, health insurance coverage, and prescription drug insurance coverage), enabling factor (presence/absence of other chronic conditions, pain interference), personal health practice (obesity, smoking status, and exercise level), and external environment (region). ^‡^Confidence intervals are based on 2000 bootstrap replications using the percentile method. ^∗^*p* < 0.05; ^∗∗^*p* < 0.01; ^∗∗∗^*p* < 0.001.

**Table 4:** Unadjusted and adjusted association between depression and anxiety categories and high out-of-pocket (OOP) spending burden among adults with comorbid diabetes and hypertension, using pooled data from the 2013 and 2015 Medical Expenditure Panel Survey.

Bivariate association between depression and anxiety categories and high OOP spending burden						
	High burden^†^		Not high burden			
	*N*	Wt row%	*N*	Wt row%	*p* value	Sig.
Depression and anxiety	90	34.0	219	66.0	<0.001	^∗∗∗^
Depression only	158	28.2	403	71.8		
Anxiety only	96	23.5	270	76.5		
No depression/anxiety	650	18.3	2674	81.7		

Logistic regressions on high OOP spending burden^†^						
	Unadjusted model			Adjusted model^‡^		
	OR	95% CI	Sig.	AOR	95% CI	Sig.
Depression and anxiety	2.26	(1.66–3.07)	^∗∗∗^	1.55	(1.06–2.25)	^∗^
Depression only	1.72	(1.28–2.32)	^∗∗∗^	1.25	(0.91–1.70)	
Anxiety only	1.34	(1.04–1.74)	^∗^	1.03	(0.77–1.38)	
No depression/anxiety	(reference group)					

Note: the analytic sample consisted of adults (>18 years) with comorbid diabetes and hypertension who were alive and had positive expenditures in the observation year (2013/2015). Wt row%: weighted row percentages; Sig.: statistical significance level; OR: odds ratio; AOR: adjusted odds ratio; 95% CI: 95% confidence interval. ^†^OOP spending burden was measured by the percentage of household income spent on healthcare. It was calculated by dividing OOP spending by household income. High OOP spending burden was defined as 10% or more. ^‡^Covariates adjusted in the logistic regression included predisposing factors (sex, age groups, and race/ethnicity), enabling factors (marital status, education level, poverty status, health insurance coverage, and prescription drug insurance coverage), enabling factor (presence/absence of other chronic conditions, pain interference), personal health practice (obesity, smoking status, and exercise level), and external environment (region). ^∗^*p* < 0.05; ^∗∗^*p* < 0.01; ^∗∗∗^*p* < 0.001.

## Data Availability

The data used to support the findings of this study are from MEPS Household Component public use data files, which are available for downloading on the MEPS website (https://meps.ahrq.gov/mepsweb/data_stats/download_data_files.jsp).
